# Case Report: Cyclophosphamide in COVID-19 – when an absolute contraindication is an absolute necessity

**DOI:** 10.12688/f1000research.55625.1

**Published:** 2021-08-19

**Authors:** Kamila Bołtuć, Ada Bielejewska, Alejandro Coloma-Millar, Robert Dziugieł, Arkadiusz Bociek, Agnieszka Perkowska-Ptasińska, Andrzej Jaroszyński

**Affiliations:** 1 Collegium Medicum, Jan Kochanowski University, Kielce, Poland; 2 Nephrology Clinic, Voivodeship Hospital, Kielce, Poland; 3 Department of Pathomorphology, Medical University of Warsaw, Warsaw, Poland

**Keywords:** COVID-19, vasculitis, alveolar haemorrhage, p-ANCA, cyclophosphamide

## Abstract

**Background: **Despite many studies on COVID-19, our knowledge of it remains incomplete. In some cases, treating SARS-CoV-2 infection concomitant with other diseases can be particularly challenging, as finding an appropriate treatment may involve some risks.

**Case presentation: **A 34-year-old SARS-CoV-2 positive patient admitted due to fever, dyspnoea, haemoptysis and pneumonia, developed alveolar haemorrhage and acute kidney injury. Due to his severe state, abnormalities in laboratory tests and rapidly progressing loss of kidney function, kidney biopsy, as well as antibody panel were carried out, in which perinuclear anti-neutrophil cytoplasmic antibodies (p-ANCA) were found with a high titer (>200; N: <1:20). The results of kidney biopsy, combined with clinical manifestation and laboratory findings prompted the diagnosis of rapidly progressing glomerulonephritis (RPGN) in the course of p-ANCA vasculitis. Initial treatment consisted of heamodialyses, remdesivir, plasmaphereses, intravenous immunoglobulins, antibiotics, corticosteroids and fraxiparine. Once the haemorrhage had subsided, kidney function had been partially retrieved and heamodialyses had no longer been necessary, cyclophosphamide treatment was initiated, despite being contraindicated in COVID-19 according to its summary of product characteristics. Immunotherapy is still continued. The patient has already received a total of 2.4g of cyclophosphamide (4 cycles of 600mg each every three weeks). Pulmonary and radiological regression, as well as improvement of renal parameters have been achieved.

**Conclusions: **We suspect that cyclophosphamide, the drug of choice in p-ANCA vasculitis, could be a potential factor providing regression of the radiological changes in the lungs and it could have prevented the patient from developing acute respiratory distress syndrome. COVID-19 diagnosis should not exclude searching for other diseases which can have a similar course. When treating a patient in a life-threatening condition, a departure from trying to find the perfect timing of cyclophosphamide delivery should be considered, as delaying it could cause potentially greater harm.

## List of abbreviations

AKI: acute kidney injury

ANA: antinuclear antibodies

anti-GBM: anti-glomerular basement membrane antibodies

ARDS: acute respiratory distress syndrome

BP: blood pressure

c-ANCA: cytoplasmic anti-neutrophil cytoplasmic antibodies

CRP: C-reactive protein

CT: chest computed tomography

IL-6: interleukin 6

IVIG: intravenous immunoglobulins

p-ANCA: perinuclear anti-neutrophil cytoplasmic antibodies

RPGN: rapidly progressing glomerulonephritis

## Introduction

More than a year has passed since COVID-19 emerged as a global pandemic. Despite many studies, our knowledge of this disease remains incomplete, especially in SARS-CoV-2 infection with concomitant diseases. Thus, many clinicians face difficulties with finding an appropriate treatment for some cases.

## Case report

A 34-year-old SARS-CoV-2-positive Caucasian male with a history of borderline hypertension, hypothyroidism and transient arthralgia 2 years prior (without any further investigation), was admitted to a pulmonology ward in November 2020 due to fever (up to 38.5°C), dyspnoea, haemoptysis and developing bilateral pneumonia. During hospitalization the patient developed alveolar haemorrhage followed by acute kidney injury (AKI), for which he was transferred to a Nephrology Clinic on November 21, 2020. On admission he was afebrile and presented with mild haemoptysis and conjunctivitis, oxygen saturation 86-94% (oxygen flow 10-15 l/min), blood pressure (BP) 120/70 mmHg, numerous erythrocytes in urine sediment and proteinuria of 0.47 g/l. His condition was severe and deteriorating.

**Figure 1.**
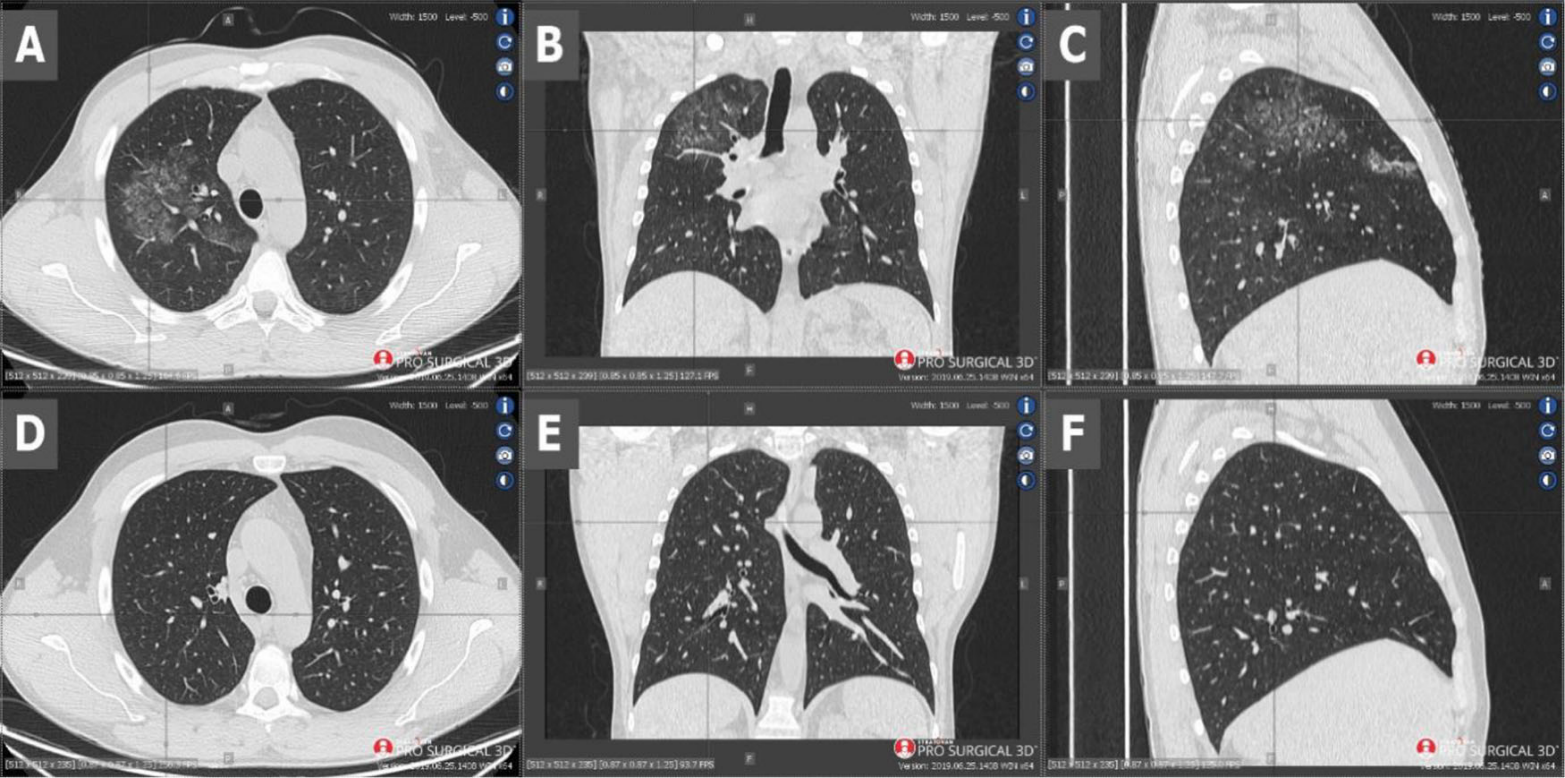
Scans A-C - CT performed on admission - scans show diffuse ground-glass opacities and septal interlobular thickening in the right lung, as well as paving-stone findings which could represent alveolar haemorrhage. Scans D-F - CT performed 2 months later - visible radiological regression.

**Figure 2.**
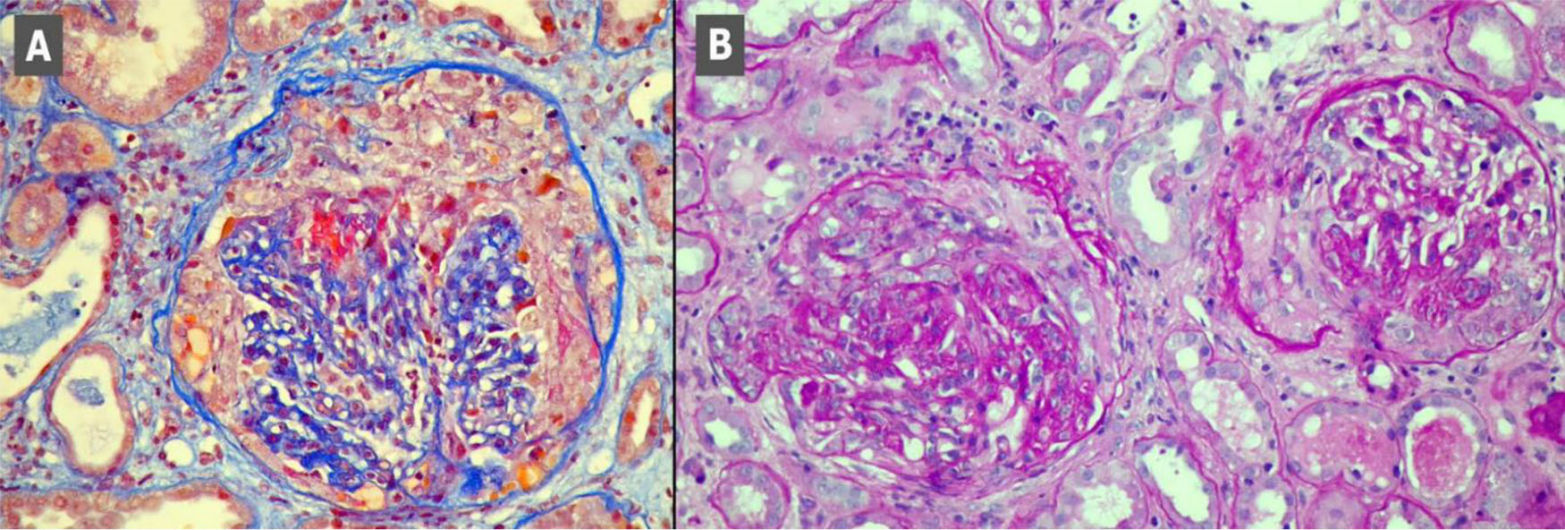
Kidney biopsy scans. Scan A - segmental necrosis and cellular crescent, Masson stain. Scan B - Glomeruli with fibrocellular crescents, PAS stain.

Owing to the anamnesis of transient arthralgia and rapid deterioration of the kidney function, antibody panel was carried out in which anti-glomerular basement membrane antibodies (anti-GBM), antinuclear antibodies (ANA), as well as cytoplasmic anti-neutrophil cytoplasmic antibodies (c-ANCA) were negative, whereas perinuclear anti-neutrophil cytoplasmic antibodies (p-ANCA) were found with a high titer (>200; N: <1:20), suggesting p-ANCA vasculitis complicating COVID-19. Laboratory tests revealed AKI requiring haemodialysis. Anaemia was particularly noticeable. Additionally, leukocytosis, hypoproteinemia, dysproteinemia, elevated D-dimers, C-reactive protein (CRP), interleukin 6 (IL-6), creatinine and urea levels, proteinuria and haematuria were present ( Table 1). The parameters of the blood coagulation system were within normal range. Chest computed tomography (CT) demonstrated changes suggesting both viral pneumonia and alveolar haemorrhage ( Figure 1, scans A-C). Kidney biopsy performed on the first day of hospitalisation in the Nephrology Clinic and examined with a light microscope revealed crescents in segmental tuft necrosis in two, cellular crescent in one and fibrocellular crescents in three out of nine glomeruli. Glomerular lesions were accompanied by mild interstitial inflammation and acute tubular necrosis. There was no interstitial fibrosis nor tubular atrophy. Immunofluorescence did not reveal any deposits of immunoglobulins nor complement components ( Figure 2). The biopsy result, combined with clinical manifestation, prompted the diagnosis of rapidly progressing glomerulonephritis (RPGN) in the course of p-ANCA vasculitis.

**Table 1.** Patient’s laboratory results on admission.

Parameter	Patient value	Normal range
Haemoglobin [g/dl]	9.3	13.7-16.5
Serum total protein [g/dl]	4.90	6.00-8.00
Alpha 1 [g/dl]	0.26	0.21-0.35
Alpha 2 [g/dl]	0.37	0.51-0.85
Beta 1 [g/dl]	0.25	0.34-0.52
Beta 2 [g/dl]	0.18	0.23-0.47
Gamma [g/dl]	0.56	0.80-1.35
D-dimer [ug/l]	2378.0	0.0-500.0
White blood cell count [K/ul]	16.20	4.23-9.07
CRP [mg/l]	35.46	0.10-5.00
IL-6 [pg/ml]	92.0	<7.0
Creatinine [mg/dl]	3.2	0.70-1.30
Urea [mg/dl]	89	20-45
Total protein in urine [g/l]	0.47	0.05-0.08
Erythrocytes in urine sediment	numerous	0-5 in visual field

In spite of haemodialysis, the patient was treated with remdesivir (0.1 g). Parallelly to remdesivir therapy, in order to stop the immunological process causing the bleeding, the patient underwent four plasmaphereses with both fresh frozen plasma and albumin as replacement fluids and citrate as anticoagulant. On account of anaemia, the patient was given four units of packed red blood cells. On the fifth and sixth day of hospitalization, a total of 40 g of intravenous immunoglobulins (IVIG) were ordered to inhibit the underlying immunological process responsible for alveolar bleeding in the course of p-ANCA vasculitis. Moreover, owing to the alveolar haemorrhage and probable bacterial superinfection, clarithromycin and piperacillin with tazobactam were ordered. Simultaneously, corticosteroid treatment was started when the results of p-ANCA antibodies were received (1.0 g of intravenous methylprednisolone for 4 consecutive days, followed by 40 mg of oral prednisone). Initially, due to alveolar bleeding, fraxiparine treatment could not be initiated, despite increasing values of D-dimers (2378.0 ug/l vs 4053.0 ug/l 3 days later; N: 0.0-500.0). It was not until after the bleeding had stopped, that it was prescribed (2× 0.6 ml). Once the haemorrhage had subsided, kidney function had been partially retrieved and heamodialyses had no longer been necessary, cyclophosphamide treatment was initiated on the eighth day of hospitalization, despite being contraindicated in COVID-19 according to its summary of product characteristics.

Currently, immunotherapy is continued. To date, the patient has received a total of 2.4 g (four cycles of 600 mg each, 3 weeks apart) of cyclophosphamide. The therapy is well-tolerated and a pulmonary clinical and radiological remission has been achieved ( Figure 1, scans D-F). Additionally, renal parameters have improved with creatinine level at 1.1 mg/dl and stable proteinuria of 1.8 g/24 h.

## Discussion

While there are cases of continuing immunosuppressive therapy during COVID-19, no data related to the onset of this treatment while undergoing the acute phase of SARS-CoV-2 infection and the follow-up has been reported yet. To our best knowledge, this is the first report of introducing the cyclophosphamide treatment during the acute phase of COVID-19 with the follow-up.

Our case presents a critically ill patient whose state was deteriorating despite the standard treatment. Even though SARS-CoV-2 has been known to induce AKI even in 22.2% of hospitalized patients, ^1^ as well as to cause pneumorrhagia, ^2^ the coincidence of renal and pulmonary symptoms prompted us to search for another reason for the patient’s state other than merely COVID-19. Confirmed p-ANCA vasculitis was an indication to introduce immunosuppressive treatment. However, an active SARS-CoV-2 infection is considered an absolute contraindication for this therapy. Even so, the treatment was still initiated, since the patient’s life was in danger and it was the last resort. We decided to try to stabilize the patient and wait till the estimated time of the viral replication phase end to introduce cyclophosphamide. ^3^

Despite the fact that COVID-19 could have been the reason for pneumorrhagia and such severe state of the patient, systemic vasculitis seemed to be the more likely cause. Thus, the limitation of vasculitis effects and induction of immunosuppression was essential. To this end, plasmapheresis and high doses of IVIG were ordered. It is worth noting that IVIG and plasmapheresis could limit a SARS-CoV-2 infection and increase the tolerance to immunosuppressive treatment ^4^ which was twice as beneficial for our patient. The patient also received remdesivir to inhibit the virus multiplication and invasion. It should be noted that starting immunosuppression in the acute phase of the disease was very risky. Surprisingly, not only did cyclophosphamide not harm the patient, but also the whole applied treatment improved the patient’s state remarkably. CT performed 32 days after the initial CT (after three cycles of cyclophosphamide) showed total regression of ground-glass opacities and septal interlobular thickening and paving-stone findings ( Figure 1, scans D-F), even though radiological changes in the lungs in COVID-19 can persist for a long time after recovering from the disease. ^5^

It seems that our case may indirectly be in line with the hypothesis about the positive influence of immunosuppressive treatment in COVID-19. ^6^ Although IVIG and plasmapheresis therapy was not meaningless, this treatment alone would not lead to remission. We suspect that it was in fact cyclophosphamide, the drug of choice in p-ANCA vasculitis, that could be a potential factor providing regression of the radiological changes in the lungs and it could have prevented the patient from developing acute respiratory distress syndrome (ARDS). It could have happened by the drug’s immunomodulatory function - the second phase of COVID-19 is strongly linked to immunological response, as innate immunity mediated damage of pneumocytes and capillary leak, brought by activation of cytotoxic and effector T-cells, take place in it. The immunomodulatory function of cyclophosphamide led to the elimination of inflammatory damages’ origin. We point out that the onset of treatment happened during the second phase of COVID-19. Having regard to the high potential of replication of SARS CoV-2, immunosuppressive treatment in the replication phase could be tragic.

## Conclusion

COVID-19 diagnosis should not exclude searching for other diseases which can have a similar course. When treating a patient in a life-threatening condition, a departure from trying to find the perfect timing of cyclophosphamide delivery should be considered, as delaying it could cause potentially greater harm.

## Declarations

Consent for publication – Written informed consent for publication was obtained from the patient.

Data availability - All data underlying the results are available as part of the article and no additional source data are required.
