# Supplementary material for: Case Report: Cyclophosphamide in COVID-19 – when an absolute contraindication is an absolute necessity
Source: F1000Res. 2021 Oct 25;10:829. Originally published 2021 Aug 19. [Version 2] doi: 10.12688/f1000research.55625.2 (PMC8436184; doi:10.12688/f1000research.55625.2)
Supplement: Peer review history

# Peer review: Reviewer response for version 2

**DOI:** 10.5256/f1000research.78504.r97919

**Published:** 2021-10-26

**Authors:** Anna Clementi

**Affiliations:** 1 Nephrology and Dialysis Department, Santa Marta and Santa Venera Hospital, Acireale, Catania, Italy

I have no other comments.

Are enough details provided of any physical examination and diagnostic tests, treatment given and outcomes?

Yes

Is the case presented with sufficient detail to be useful for other practitioners?

Yes

Is sufficient discussion included of the importance of the findings and their relevance to future understanding of disease processes, diagnosis or treatment?

Yes

Is the background of the case’s history and progression described in sufficient detail?

Yes

Reviewer Expertise:

Nephrology

I confirm that I have read this submission and believe that I have an appropriate level of expertise to confirm that it is of an acceptable scientific standard.

# Peer review: Reviewer response for version 1

**DOI:** 10.5256/f1000research.59218.r93992

**Published:** 2021-09-10

**Authors:** Krzysztof J. Filipiak

**Affiliations:** 1 1st Department of Cardiology, Medical University of Warsaw, Warsaw, Poland

A very interesting case report describing the possibility of cyclophosphamide use in COVID-19. Immunosuppressive treatment in COVID-19 is a question of discussion. Cyclophosphamide, the drug of choice in p-ANCA vasculitis, could be a potential factor providing regression of the radiological changes in the lungs and it could have prevented the patient from developing acute respiratory distress syndrome (ARDS). The case is well illustrated and well written. As the immunomodulatory function of cyclophosphamide led to the elimination of inflammatory damages’ origin, broader studies are needed. 

 One thing requiring change: there is no such drug as "fraxiparine" - do the authors mean nadroparin?

Are enough details provided of any physical examination and diagnostic tests, treatment given and outcomes?

Yes

Is the case presented with sufficient detail to be useful for other practitioners?

Yes

Is sufficient discussion included of the importance of the findings and their relevance to future understanding of disease processes, diagnosis or treatment?

Yes

Is the background of the case’s history and progression described in sufficient detail?

Yes

Reviewer Expertise:

covid-19

I confirm that I have read this submission and believe that I have an appropriate level of expertise to confirm that it is of an acceptable scientific standard.

# Response

**Published:** 2021-10-03

**Authors:** Kamila Bołtuć

**Affiliations:** Jan Kochanowski University, Poland

Thank you so much for your review. In this particular case, we wanted to highlight the complexity of treatment in COVID-19 patients and present the difficulties associated with it. We are glad that this case report met your expectations and may be useful for other clinical practitioners. Additionally, thank you for finding an error with the drug. It should be Nadroparin and it will be changed.

# Peer review: Reviewer response for version 1

**DOI:** 10.5256/f1000research.59218.r92236

**Published:** 2021-09-03

**Authors:** Anna Clementi

**Affiliations:** 1 Nephrology and Dialysis Department, Santa Marta and Santa Venera Hospital, Acireale, Catania, Italy

This case report is very well described. Both the patient's clinical history and progression are presented in a detailed way, as well as diagnostic tests and outcomes.

 This is a case of p-ANCA (perinuclear anti-neutrophil cytoplasmic antibodies) vasculitis complicating COVID-19 in a 34-year-old Caucasian male with a history of borderline hypertension, hypothyroidism, and transient arthralgia 2 years prior admission.

 The patient was admitted to the hospital because of fever, dyspnoea, haemoptysis, and bilateral pneumonia. Hospitalization was then complicated by acute kidney injury and alveolar haemorrhage. Due to his clinical history and progression, an antibody panel was carried out and a high titer of p-ANCA was found, suggesting another disease in addition to COVID-19. Chest computed tomography (CT) demonstrated the presence of both viral pneumonia and alveolar haemorrhage. A kidney biopsy revealed a rapidly progressing glomerulonephritis in the course of p-ANCA vasculitis. The patient was treated with remdesivir, antibiotics, corticosteroids, IV immunoglobulins, and hemodialysis, and plasmaphereses were performed as well. Also, cyclophosphamide treatment was initiated, despite being contraindicated in the case of COVID-19.

 First of all, this case report highlights the importance of considering other diseases which can have a similar course to COVID-19. Furthermore, the choice of each treatment is reported in a reasonable and clear way. In particular, the introduction of immunosuppressive treatment with cyclophosphamide may have improved the patient’s clinical condition by modifying the immune response to SARS-CoV-2.

 I think this is a very interesting case report which can be very useful for clinicians, especially during this period of pandemic.

Are enough details provided of any physical examination and diagnostic tests, treatment given and outcomes?

Yes

Is the case presented with sufficient detail to be useful for other practitioners?

Yes

Is sufficient discussion included of the importance of the findings and their relevance to future understanding of disease processes, diagnosis or treatment?

Yes

Is the background of the case’s history and progression described in sufficient detail?

Yes

Reviewer Expertise:

Nephrology

I confirm that I have read this submission and believe that I have an appropriate level of expertise to confirm that it is of an acceptable scientific standard.

# Response

**Published:** 2021-10-02

**Authors:** Kamila Bołtuć

**Affiliations:** Jan Kochanowski University, Poland

Thank you so much for this detailed review. 

 We tried to highlight the possibility of the COVID-19 concomitance with various diseases. Moreover, based on this case we wanted to demonstrate the complexity of treatment in COVID-19 patients. 

 We are glad that our case report met your expectations and due to sufficient detail may be useful for other clinicians.
